# URINARY INCONTINENCE AS A RISK TO PHYSICAL, MENTAL, AND SOCIAL HEALTH AMONG OLDER KOREAN AMERICANS IN SENIOR HOUSING

**DOI:** 10.1093/geroni/igae098.3350

**Published:** 2024-12-31

**Authors:** Juyoung Park, Yuri Jang

**Affiliations:** USC Suzanne Dworak-Peck School of Social Work, Los Angeles, California, United States; University of Southern California, Los Angeles, California, United States

## Abstract

Urinary incontinence (UI) or involuntary leakage of urine, is a common geriatric condition. Despite its harmful effect on health and well-being, UI has often been neglected in geriatric care. In the present study, we examined the risks posed by UI to the physical, mental, and social health of older Korean Americans living in subsidized senior housing. Survey data were collected from 313 older Korean-American residents in subsidized senior housing in the Los Angeles area. UI was assessed with a question on the frequency of experiencing involuntary leakage of urine. Physical, mental, and social health risks were indicated by a single item self-rated health (fair/poor rating), the Patient Health Questionnaire−9 (probable depression), and the Lubben Social Network Scale−6, (isolation from family and isolation from friends). More than half of the sample reported UI, with the experience being infrequent (i.e., seldom) for 46.3% and frequent (i.e., sometimes or often) for 10.3%. Regardless of the frequency, UI posed a significant risk to physical and mental health. The odds of reporting fair/poor health and having probable depression were 1.82 to 6.81 times higher among those with infrequent/frequent UI than among those without. Family isolation was not associated with UI; however, the odds of being isolated from friends were 2.95 times higher among those with frequent UI compared with the reference group. Our findings underscore the importance of addressing UI in the health promotion effort in senior housing, calling for interdisciplinary approaches for resident care.

